# Supplementary material for: Impact of urbanization on functional diversity in macromycete communities along an urban ecosystem in Southwest Mexico
Source: PeerJ. 2021 Sep 21;9:e12191. doi: 10.7717/peerj.12191 (PMC8462387; doi:10.7717/peerj.12191)
Supplement: Supplemental Information 1 — “sp., sp. 1, sp. 2, sp. 3…” indicate species unidentified (morphospecies). The functional guilds (FG) are ectomycorrhizas (E), saprotrophs (S), and parasites (P). [file peerj-09-12191-s001.docx]

Macromycete species, Families, Orders, Classes and Phyla recorded within the studied area at Southwest, Mexico. “sp., sp. 1, sp. 2, sp. 3…” indicate species unidentified (morphospecies). The functional guilds (FG) are ectomycorrhizas (E), saprotrophs (S), and parasites (P).

| **Species** | **Family** | **Order** | **Class** | **Phylum** | **FG** |
| --- | --- | --- | --- | --- | --- |
| *Agaricus* sp. 1 | Agaricaceae | Agaricales | Agaricomycetes | Basidiomycota | S |
| *Agaricus* sp. 2 | Agaricaceae | Agaricales | Agaricomycetes | Basidiomycota | S |
| *Agaricus* sp. 3 | Agaricaceae | Agaricales | Agaricomycetes | Basidiomycota | S |
| *Agaricus* sp. 4 | Agaricaceae | Agaricales | Agaricomycetes | Basidiomycota | S |
| *Agaricus* sp. 5 | Agaricaceae | Agaricales | Agaricomycetes | Basidiomycota | S |
| *Amanita* aff. *constricta* | Amanitaceae | Agaricales | Agaricomycetes | Basidiomycota | E |
| *Amanita* aff. *fulva* | Amanitaceae | Agaricales | Agaricomycetes | Basidiomycota | E |
| *Amanita gemmata* (Fr.) Bertill. | Amanitaceae | Agaricales | Agaricomycetes | Basidiomycota | E |
| *Amanita* aff. *gemmata* | Amanitaceae | Agaricales | Agaricomycetes | Basidiomycota | E |
| *Amanita muscaria* (L.) Lam. | Amanitaceae | Agaricales | Agaricomycetes | Basidiomycota | E |
| *Amanita vaginata* (Bull.) Lam. | Amanitaceae | Agaricales | Agaricomycetes | Basidiomycota | E |
| *Amanita* sp.1 | Amanitaceae | Agaricales | Agaricomycetes | Basidiomycota | E |
| *Amanita* sp.2 | Amanitaceae | Agaricales | Agaricomycetes | Basidiomycota | E |
| *Amanita* sp.3 | Amanitaceae | Agaricales | Agaricomycetes | Basidiomycota | E |
| *Amanita* sp.4 | Amanitaceae | Agaricales | Agaricomycetes | Basidiomycota | E |
| *Amanita* sp.5 | Amanitaceae | Agaricales | Agaricomycetes | Basidiomycota | E |
| *Annulohypoxylon truncatum* (Starbäck) Y.M. Ju, J.D. Rogers & H.M. Hsieh | Hypoxylaceae | Xylariales | Sordariomycetes | Ascomycota | S |
| *Annulohypoxylo*n sp.1 | Hypoxylaceae | Xylariales | Sordariomycetes | Ascomycota | S |
| *Annulohypoxylon* sp.2 | Hypoxylaceae | Xylariales | Sordariomycetes | Ascomycota | S |
| *Annulohypoxylon* sp.3 | Hypoxylaceae | Xylariales | Sordariomycetes | Ascomycota | S |
| *Annulohypoxylon thouarsianum* (Lév.) Y.M. Ju, J.D. Rogers & H.M. Hsieh | Hypoxylaceae | Xylariales | Sordariomycetes | Ascomycota | S |
| *Armillaria* sp.1 | Physalacriaceae | Agaricales | Agaricomycetes | Basidiomycota | P |
| *Austroboletus* aff. *subflavidus* | Boletaceae | Boletales | Agaricomycetes | Basidiomycota | E |
| *Biscogniauxia atropunctata* (Schwein.) Pouzar | Graphostromataceae | Xylariales | Sordariomycetes | Ascomycota | S |
| *Boletus* aff. *edulis* | Boletaceae | Boletales | Agaricomycetes | Basidiomycota | E |
| *Boletus* aff. *singeri* | Boletaceae | Boletales | Agaricomycetes | Basidiomycota | E |
| *Boletus* sp.1 | Boletaceae | Boletales | Agaricomycetes | Basidiomycota | E |
| *Boletus* sp.2 | Boletaceae | Boletales | Agaricomycetes | Basidiomycota | E |
| *Boletus* sp.3 | Boletaceae | Boletales | Agaricomycetes | Basidiomycota | E |
| *Boletus* sp.4 | Boletaceae | Boletales | Agaricomycetes | Basidiomycota | E |
| *Boletus* sp.5 | Boletaceae | Boletales | Agaricomycetes | Basidiomycota | E |
| *Boletus* sp.6 | Boletaceae | Boletales | Agaricomycetes | Basidiomycota | E |
| *Boletus* sp.7 | Boletaceae | Boletales | Agaricomycetes | Basidiomycota | E |
| *Boletus variipes* A.H. Sm. & Thiers | Boletaceae | Boletales | Agaricomycetes | Basidiomycota | E |
| *Byssothecium* sp.1 | Massarinaceae | Pleosporales | Dothideomycetes | Ascomycota | S |
| *Calocybe* sp.1 | Lyophyllaceae | Agaricales | Agaricomycetes | Basidiomycota | S |
| *Cantharellus cibarius* Fr. | Cantharellaceae | Cantharellales | Agaricomycetes | Basidiomycota | E |
| *Cantharellus* sp.1 | Cantharellaceae | Cantharellales | Agaricomycetes | Basidiomycota | E |
| *Clitocybe* sp.1 | Tricholomataceae | Agaricales | Agaricomycetes | Basidiomycota | S |
| *Coprinellus* sp.1 | Psathyrellaceae | Agaricales | Agaricomycetes | Basidiomycota | S |
| *Coprinellus* sp.2 | Psathyrellaceae | Agaricales | Agaricomycetes | Basidiomycota | S |
| *Coprinus* sp.1 | Agaricaceae | Agaricales | Agaricomycetes | Basidiomycota | S |
| *Cortinarius* sp.1 | Cortinariaceae | Agaricales | Agaricomycetes | Basidiomycota | E |
| *Cortinarius* sp.2 | Cortinariaceae | Agaricales | Agaricomycetes | Basidiomycota | E |
| *Cortinarius* sp.3 | Cortinariaceae | Agaricales | Agaricomycetes | Basidiomycota | E |
| *Cortinarius* sp.4 | Cortinariaceae | Agaricales | Agaricomycetes | Basidiomycota | E |
| *Cortinarius* sp.5 | Cortinariaceae | Agaricales | Agaricomycetes | Basidiomycota | E |
| *Cortinarius* sp.6 | Cortinariaceae | Agaricales | Agaricomycetes | Basidiomycota | E |
| *Cortinarius* sp.7 | Cortinariaceae | Agaricales | Agaricomycetes | Basidiomycota | E |
| *Cortinarius* sp.8 | Cortinariaceae | Agaricales | Agaricomycetes | Basidiomycota | E |
| *Cortinarius* sp.9 | Cortinariaceae | Agaricales | Agaricomycetes | Basidiomycota | E |
| *Diatrypella favacea* (Fr.) Ces. & De Not. | Diatrypaceae | Xylariales | Sordariomycetes | Ascomycota | S |
| *Entoloma* sp.1 | Entolomataceae | Agaricales | Agaricomycetes | Basidiomycota | S |
| *Eutypella tumida* (Ellis & Everh.) Wehm. | Diatrypaceae | Xylariales | Sordariomycetes | Ascomycota | P |
| *Fomitiporia* sp.1 | Hymenochaetaceae | Hymenochaetales | Agaricomycetes | Basidiomycota | P |
| *Glonium* sp.1 | Gloniaceae | Mytilinidiales | Dothideomycetes | Ascomycota | S |
| *Gymnopus* aff. *peronatus* | Omphalotaceae | Agaricales | Agaricomycetes | Basidiomycota | S |
| *Gymnopus* sp.1 | Omphalotaceae | Agaricales | Agaricomycetes | Basidiomycota | S |
| *Helvella acetabulum* (L.) Quél. | Helvellaceae | Pezizales | Pezizomycetes | Ascomycota | E |
| *Helvella crispa* (Scop.) Fr. | Helvellaceae | Pezizales | Pezizomycetes | Ascomycota | E |
| *Helvella fusca* Gillet | Helvellaceae | Pezizales | Pezizomycetes | Ascomycota | E |
| *Helvella macropus* (Pers.) P. Karst. | Helvellaceae | Pezizales | Pezizomycetes | Ascomycota | E |
| *Humaria hemisphaerica* (F.H. Wigg.) Fuckel | Pyronemataceae | Pezizales | Pezizomycetes | Ascomycota | S |
| *Hydnellum aurantiacum* (Batsch) P. Karst. | Bankeraceae | Thelephorales | Agaricomycetes | Basidiomycota | E |
| *Hypoxylon* sp.1 | Hypoxylaceae | Xylariales | Sordariomycetes | Ascomycota | S |
| *Hypoxylon* sp.2 | Hypoxylaceae | Xylariales | Sordariomycetes | Ascomycota | S |
| *Hypoxylon* sp.3 | Hypoxylaceae | Xylariales | Sordariomycetes | Ascomycota | S |
| *Hysterium* sp.1 | Hysteriaceae | Hysteriales | Dothideomycetes | Ascomycota | S |
| *Laccaria* sp.1 | Hydnangiaceae | Agaricales | Agaricomycetes | Basidiomycota | E |
| *Laccaria* sp.2 | Hydnangiaceae | Agaricales | Agaricomycetes | Basidiomycota | E |
| *Lachnum* sp.1 | Lachnaceae | Helotiales | Leotiomycetes | Ascomycota | S |
| *Lactarius azonites* (Bull.) Fr. | Russulaceae | Russulales | Agaricomycetes | Basidiomycota | E |
| *Lactarius indigo* (Schwein.) Fr. | Russulaceae | Russulales | Agaricomycetes | Basidiomycota | E |
| *Lactarius piperatus* (L.) Pers. | Russulaceae | Russulales | Agaricomycetes | Basidiomycota | E |
| *Lactarius resimus* (Fr.) Fr. | Russulaceae | Russulales | Agaricomycetes | Basidiomycota | E |
| *Lactarius* sp.1 | Russulaceae | Russulales | Agaricomycetes | Basidiomycota | E |
| *Lactarius* sp.2 | Russulaceae | Russulales | Agaricomycetes | Basidiomycota | E |
| *Lactarius* sp.3 | Russulaceae | Russulales | Agaricomycetes | Basidiomycota | E |
| *Lactarius* sp.4 | Russulaceae | Russulales | Agaricomycetes | Basidiomycota | E |
| *Lactarius* sp.5 | Russulaceae | Russulales | Agaricomycetes | Basidiomycota | E |
| *Lactarius volemus* (Fr.) Fr. | Russulaceae | Russulales | Agaricomycetes | Basidiomycota | E |
| *Lepiota pseudolilacea* Huijsman | Agaricaceae | Agaricales | Agaricomycetes | Basidiomycota | S |
| *Lepiota* sp.1 | Agaricaceae | Agaricales | Agaricomycetes | Basidiomycota | S |
| *Leucoagaricus* sp.1 | Agaricaceae | Agaricales | Agaricomycetes | Basidiomycota | S |
| *Lycoperdon perlatum* Pers. | Agaricaceae | Agaricales | Agaricomycetes | Basidiomycota | S |
| *Lycoperdon* sp.1 | Agaricaceae | Agaricales | Agaricomycetes | Basidiomycota | S |
| *Lycoperdon* sp.2 | Agaricaceae | Agaricales | Agaricomycetes | Basidiomycota | S |
| *Lycoperdon* sp.3 | Agaricaceae | Agaricales | Agaricomycetes | Basidiomycota | S |
| *Lycoperdon* sp.4 | Agaricaceae | Agaricales | Agaricomycetes | Basidiomycota | S |
| *Mycena* sp.1 | Mycenaceae | Boletales | Agaricomycetes | Basidiomycota | S |
| *Mycena* sp.2 | Mycenaceae | Boletales | Agaricomycetes | Basidiomycota | S |
| *Mycetinis* aff. s*corodonius* | Omphalotaceae | Agaricales | Agaricomycetes | Basidiomycota | S |
| *Peniophora quercina* (Pers.) Cooke | Peniophoraceae | Russulales | Agaricomycetes | Basidiomycota | S |
| *Peziza violacea* Pers. | Pezizaceae | Pezizales | Pezizomycetes | Ascomycota | S |
| *Phellinus* aff. *gilvus* | Hymenochaetaceae | Hymenochaetales | Agaricomycetes | Basidiomycota | P |
| *Phellinus pomaceus* (Pers.) Maire | Hymenochaetaceae | Hymenochaetales | Agaricomycetes | Basidiomycota | P |
| *Plectania rhytidia* (Berk.) Nannf. & Korf | Sarcosomataceae | Pezizales | Pezizomycetes | Ascomycota | S |
| *Polyporus* sp.1 | Polyporaceae | Polyporales | Agaricomycetes | Basidiomycota | S |
| *Polyporus* sp.2 | Polyporaceae | Polyporales | Agaricomycetes | Basidiomycota | S |
| *Ramaria* sp.1 | Gomphaceae | Gomphales | Agaricomycetes | Basidiomycota | E |
| *Ramaria* sp.2 | Gomphaceae | Gomphales | Agaricomycetes | Basidiomycota | E |
| *Rhytidhysteron* sp.1 | Patellariaceae | Patellariales | Dothideomycetes | Ascomycota | S |
| *Rosellinia* sp.1 | Xylariaceae | Xylariales | Sordariomycetes | Ascomycota | P |
| *Russula* aff. *adusta* | Russulaceae | Russulales | Agaricomycetes | Basidiomycota | E |
| *Russula* aff. *farinipes* | Russulaceae | Russulales | Agaricomycetes | Basidiomycota | E |
| *Russula* aff. *sanguinaria* | Russulaceae | Russulales | Agaricomycetes | Basidiomycota | E |
| *Russula cyanoxantha* (Schaeff.) Fr. | Russulaceae | Russulales | Agaricomycetes | Basidiomycota | E |
| *Russula* sp.1 | Russulaceae | Russulales | Agaricomycetes | Basidiomycota | E |
| *Russula* sp.2 | Russulaceae | Russulales | Agaricomycetes | Basidiomycota | E |
| *Russula* sp.3 | Russulaceae | Russulales | Agaricomycetes | Basidiomycota | E |
| *Russula* sp.4 | Russulaceae | Russulales | Agaricomycetes | Basidiomycota | E |
| *Russula* sp.5 | Russulaceae | Russulales | Agaricomycetes | Basidiomycota | E |
| *Russula* sp.6 | Russulaceae | Russulales | Agaricomycetes | Basidiomycota | E |
| *Russula* sp.7 | Russulaceae | Russulales | Agaricomycetes | Basidiomycota | E |
| *Russula* sp.8 | Russulaceae | Russulales | Agaricomycetes | Basidiomycota | E |
| *Russula* sp.9 | Russulaceae | Russulales | Agaricomycetes | Basidiomycota | E |
| *Schizophyllum commune* Fr. | Schizophyllaceae | Agaricales | Agaricomycetes | Basidiomycota | S |
| *Scleroderma* sp.1 | Sclerodermataceae | Boletales | Agaricomycetes | Basidiomycota | E |
| *Scleroderma* sp.2 | Sclerodermataceae | Boletales | Agaricomycetes | Basidiomycota | E |
| *Scleroderma* sp.3 | Sclerodermataceae | Boletales | Agaricomycetes | Basidiomycota | E |
| *Scleroderma verrucosum* (Bull.) Pers. | Sclerodermataceae | Boletales | Agaricomycetes | Basidiomycota | E |
| *Stereum ostrea* (Blume & T. Nees) Fr. | Stereaceae | Russulales | Agaricomycetes | Basidiomycota | S |
| *Stereum* sp.1 | Stereaceae | Russulales | Agaricomycetes | Basidiomycota | S |
| *Strobilomyces strobilaceus* (Scop.) Berk. | Boletaceae | Boletales | Agaricomycetes | Basidiomycota | E |
| *Suillus* sp.1 | Boletaceae | Boletales | Agaricomycetes | Basidiomycota | E |
| *Tremella* sp.1 | Tremellaceae | Tremellales | Tremellomycetes | Basidiomycota | S |
| *Tricholoma* sp.1 | Tricholomataceae | Agaricales | Agaricomycetes | Basidiomycota | E |
| *Tricholoma* sp.2 | Tricholomataceae | Agaricales | Agaricomycetes | Basidiomycota | E |
| *Tricholoma equestre* (L.) P. Kumm. | Tricholomataceae | Agaricales | Agaricomycetes | Basidiomycota | E |
| *Tylopilus* sp.1 | Boletaceae | Boletales | Agaricomycetes | Basidiomycota | E |
| *Xylaria* sp.1 | Xylariaceae | Xylariales | Sordariomycetes | Ascomycota | S |
| *Xylaria* sp.2 | Xylariaceae | Xylariales | Sordariomycetes | Ascomycota | S |
| *Xylaria* aff. *arbuscula* | Xylariaceae | Xylariales | Sordariomycetes | Ascomycota | S |
| *Xylaria hypoxylon* (L.) Grev. | Xylariaceae | Xylariales | Sordariomycetes | Ascomycota | S |
