# Supplementary figures and images for: Impact of urbanization on functional diversity in macromycete communities along an urban ecosystem in Southwest Mexico
Source: PeerJ. 2021 Sep 21;9:e12191. doi: 10.7717/peerj.12191 (PMC8462387; doi:10.7717/peerj.12191)

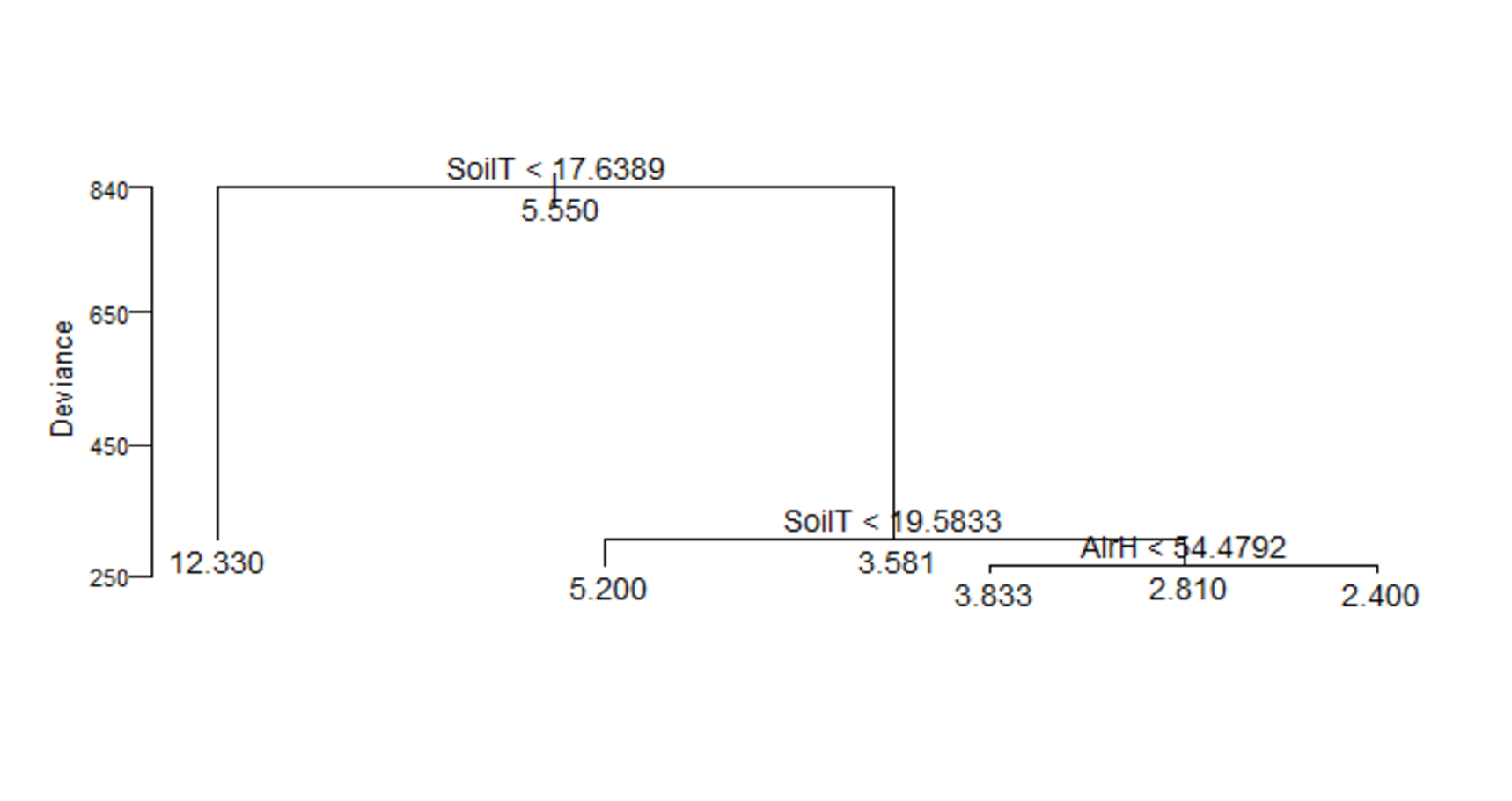

Supplement: Supplemental Information 2 — Each partition shows the explanatory variable and threshold at which the partition was made. The average value of the variable effect is indicated at the tips and nodes. Variables considered are soil temperature (SoilT) and air humidity (AirH). [file peerj-09-12191-s002.png]
