# Supplementary material for: Impact of urbanization on functional diversity in macromycete communities along an urban ecosystem in Southwest Mexico
Source: PeerJ. 2021 Sep 21;9:e12191. doi: 10.7717/peerj.12191 (PMC8462387; doi:10.7717/peerj.12191)
Supplement: Supplemental Information 3 — Matrix indicating similarity between study sites based on species composition. [file peerj-09-12191-s003.docx]

Chao-Jaccard similarity index. Matrix indicating similarity between study sites based on species composition.

Site 1 Site 2 Site 3 Site 4

Site 1 1 0.106 0.056 0.113

Site 2 1 0.075 0.163

Site 3 1 0.249

Site 4 1
