# Supplementary material for: Impact of urbanization on functional diversity in macromycete communities along an urban ecosystem in Southwest Mexico
Source: PeerJ. 2021 Sep 21;9:e12191. doi: 10.7717/peerj.12191 (PMC8462387; doi:10.7717/peerj.12191)
Supplement: Supplemental Information 4 [file peerj-09-12191-s004.docx]

Functional-value index (FV), macromycete species richness (MacroM), and number of ectomycorrhizal (EcM), saprotrophic (Sap), and pathogenic (Pat) fungal species observed in each of the 10 plots of the four study sites.

Sites Plots FV MacroM EcM Sap Pat

1 14.03 14 7 7 0

2 15.27 16 11 5 0

3 12.59 9 6 3 0

4 15.22 17 9 7 1

1 5 13.62 13 6 6 1

6 12.5 10 5 5 0

7 12.5 10 5 5 0

8 9.74 5 4 0 1

9 7.20 3 1 2 0

10 11.9 8 5 2 1

1 9.45 4 3 1 0

2 7.58 4 1 3 0

3 7.58 4 1 3 0

4 9.2 5 2 3 0

2 5 10.87 6 4 2 0

6 5.11 4 0 3 1

7 5.77 1 1 0 0

8 7.2 3 1 2 0

9 10.17 5 5 0 0

10 8.83 4 2 2 0

Sites Plots FV MacroM ECM SAP PAT

1 9.45 4 3 1 0

2 3.9 1 0 1 0

3 9.45 4 3 1 0

4 5.77 1 1 0 0

3 5 5.77 1 1 0 0

6 8.6 4 2 1 1

7 7.39 2 2 0 0

8 5.77 1 1 0 0

9 4.81 3 0 3 0

10 8.55 3 3 0 0

1 7.2 3 1 2 0

2 7.39 2 2 0 0

3 9.98 5 3 2 0

4 8.3 3 2 1 0

4 5 5.77 1 1 0 0

6 7.39 2 2 0 0

7 8.3 3 2 1 0

8 7.57 4 1 3 0

9 7.2 3 1 2 0

10 4.81 3 0 3 0
